# 

**Published:** 1886-01

**Authors:** 


### CONTENTS.

ORIGINAL COMMUNICATIONS—The Climatic Treatment of Disease—Differentiation Between Chancre and Chancroid—Revelations by Electricity—A Midwife's Diagnosis—Milk in Fevers—Y. M. D's Soliloquy .......... 299–310

ABSTRACTS FROM FOREIGN EXCHANGES .......... 320

CORRESPONDENCE—"Homœopathy" a Trade Mark—Our New York Letter .......... 322–324

SOCIETY NOTES—Physicians' Mutual Benefit Association of Texas—Death Benefit—American Public Health Association—Texas State Medical Association .......... 325–329

EDITORIAL—Smallpox in Texas—A Parallel—Dr. John H. Rauch of Illinois—Regulating the Practice—Ninth International Congress—The Amende—A Good Appointment—Married—Died .......... 333–339

ADVERTISERS' NOTICES .......... 340

